# Realizing Room‐Temperature Resonant Tunnel Magnetoresistance in Cr/Fe/MgAl_2_O_4_ Quasi‐Quantum Well Structures

**DOI:** 10.1002/advs.201901438

**Published:** 2019-08-10

**Authors:** Qingyi Xiang, Hiroaki Sukegawa, Mohamed Belmoubarik, Muftah Al‐Mahdawi, Thomas Scheike, Shinya Kasai, Yoshio Miura, Seiji Mitani

**Affiliations:** ^1^ Research Center for Magnetic and Spintronic Materials National Institute for Materials Science (NIMS) Tsukuba 305‐0047 Japan; ^2^ Graduate School of Pure and Applied Sciences University of Tsukuba Tsukuba 305‐8577 Japan; ^3^ Electrical Engineering and Electronics Kyoto Institute of Technology Kyoto 606‐8585 Japan; ^4^ Center for Materials Research by Information Integration National Institute for Materials Science (NIMS) Tsukuba 305‐0047 Japan; ^5^ Center for Spintronics Research Network (CSRN) Graduate School of Engineering Science Osaka University Osaka 560‐8531 Japan

**Keywords:** coherent tunneling, quantum well, resonant tunneling, spinel, tunnel magnetoresistance

## Abstract

The quantum well (QW) realizes new functionalities due to the discrete electronic energy levels formed in the well‐shaped potential. Magnetic tunnel junctions (MTJs) combined with a quasi‐QW structure of Cr/ultrathin‐Fe/MgAl_2_O_4_(001)/Fe, in which the Cr quasi‐barrier layer confines *Δ*_1_ up‐spin electrons to the Fe well, are prepared with perfectly lattice‐matched interfaces and atomic layer number control. Resonant peaks are clearly observed in the differential conductance of the MTJs due to the formation of QWs. Furthermore, enhanced tunnel magnetoresistance (TMR) peaks at the resonant bias voltages are realized for the MTJs at room temperature, i.e., it is observed that TMR ratios at specific and even high bias‐voltages (*V*_bias_) are larger than zero‐bias TMR ratios for the MTJs with odd Fe atomic layers, in contrast to the earlier experimental studies. In addition, a new finding in this study is unique sign changes in the temperature coefficient of resistance (TCR) depending on the Fe thickness and *V*_bias_, which is interpreted as a signature of the QW formation of Δ_1_ symmetry electronic states. The present study suggests that the spin‐dependent resonant tunneling via the QWs formed in Cr/ultrathin‐Fe/MgAl_2_O_4_/Fe structures should open a new pathway to achieve a large TMR at practically high *V*_bias_.

## Graphical abstract

**Inside Cr/ultrathin‐Fe/MgAl_2_O_4_/Fe‐based magnetic tunnel junctions, quantum well (QW) states** are established within a precisely thickness‐controlled ultrathin Fe layer. Via the QW states, the tunnel magnetoresistance (TMR) is strongly modulated at room temperature. For odd Fe atomic layer cases, the resonance appears at a high bias voltage, where the TMR is even higher than the value at zero bias.

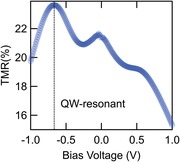

## 1 Introduction

Tunnel magnetoresistance (TMR) is the key phenomenon to achieve high‐performance spintronic devices,^1^, ^2^ e.g., magnetic random access memory (MRAM), which is of growing importance for a variety of next‐generation information processing technologies. The magnetic tunnel junctions (MTJs) consisting of ferromagnetic electrodes and an oxide barrier are widely investigated for the improvement or manipulation of TMR characteristics.^3^, ^4^, ^5^, ^6^, ^7^, ^8^, ^9^ One of the remarkable transport properties found in the MTJs is interplay of TMR effect and resonant tunneling through quantum well (QW) states,^10^, ^11^, ^12^, ^13^, ^14^, ^15^, ^16^, ^17^, ^18^, ^19^, ^20^, ^21^, ^22^, ^23^, ^24^, ^25^, ^26^ which can be realized by double‐barrier structures or simply by metallic stacking structures with a symmetry‐dependent band structure. For the ultrathin‐Fe/oxide with a (001) crystallographic orientation structure grown on a Cr buffer layer, spin‐dependent QW states are formed for the Δ_1↑_ symmetry electronic states in Fe, in which the “quasi” QW potential arises from the band mismatch between Cr Δ_1_ and Fe Δ_1↑_.^13^, ^21^, ^22^, ^23^, ^24^ It was theoretically predicted that the introduced QWs can generate resonant states at well‐defined energy levels, giving rise to giant modulation of TMR.^13^ This fact is well known as spin‐dependent resonant tunneling (SDRT) effect that can develop new functionalities. However, the experimental results reported for Cr/Fe/MgO/Fe “quasi” QW‐MTJs so far have suggested only a slight sign of the enhanced TMR at resonant bias voltages (*V*_bias_).^21^ Even for Fe/MgO/Fe/MgO/Au double‐barrier MTJs, the enhanced TMR was observed only at very low temperature.^20^

In this study, Cr/ultrathin‐Fe/MgAl_2_O_4_/Fe‐based MTJs were prepared with precise control of the ultrathin Fe layer thickness *t*_Fe_, i.e., the QW width. Owing to the perfect lattice matching between Fe and MgAl_2_O_4_,^27^, ^28^ a MgAl_2_O_4_ barrier–based double‐barrier MTJ has been reported to exhibit better QW states comparing with a MgO barrier.^26^ Associated with exact Fe layer numbers, the ultrathin‐Fe layer exhibited two almost perfect interfaces to form QWs. Hence, the SDRT effect dominating the coherent tunneling process of Δ_1_‐state electrons was clearly observed with a strong modification of TMR even at room temperature (RT). The TMR ratio at resonant *V*_bias_, at which the Δ_1↑_ channels are localized, is definitely larger than that at zero bias. In addition, it was found that due to the Δ_1↑_ states localized at specific energy levels, i.e., the formation of well‐defined QWs, positive and negative *T* dependence of tunneling conductance were distinguishably observed, depending on whether the conductance channels involve the Δ_1↑_–QW state.

Besides the fundamental understanding of the enhanced TMR, it is also important to achieve improved high‐bias performance of MTJs, i.e., large voltage output, *V*_output_ ≡ (*R*_AP_ − *R*_P_)/*R*_AP_ × *V*_bias_, particularly for magnetic sensing applications. In fact, conventional MTJs are likely to face a serious problem of the TMR degradation at high *V*_bias_. For example, while a large RT TMR ratio around 250% near zero bias in CoFeB/MgO‐based perpendicularly magnetized MTJs (p‐MTJs) was reported, it drastically drops to ≈70% at 0.5 V bias voltage, and could not survive for over 1 V.^29^ The present study offers a possible prescription to this problem, where TMR ratio at −1 V bias is almost kept same level as at zero bias.

## 2 Results and Discussion

**Figure 1:**
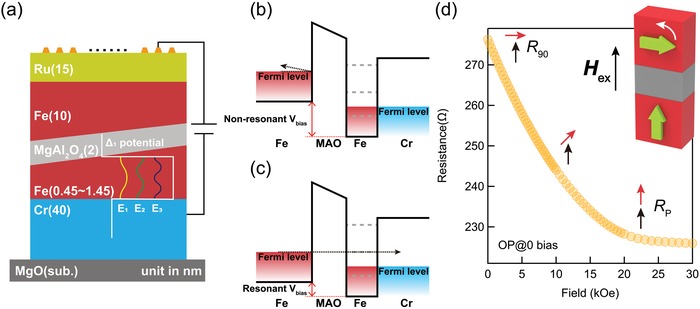
a) Schematic illustration of the thin film stack and the created quantum well potential profile. Illustrations of tunnel behavior in the quantum well at the b) nonresonant and c) resonant bias voltages, with the potential profile for Δ_1_↑ electrons. d) Out‐of‐plane field (*H*_ex_) magnetoresistance curve for an MTJ with *n*_Fe_ = 5 ML and with the orthogonal magnetization configuration. Green arrows in the inset of panel (d) indicate the easy axis of magnetization.

**Figure** **1** illustrates the MTJ stack design and the concept of SDRT via Fe QWs. To simplify the MTJ structure, MgO(001) single crystalline substrate (sub.)/Cr (40)/Fe (*t*_Fe_)/MgAl_2_O_4_ (2)/Fe (10) stacks (units in nm; *t*_Fe_ = 0.45–1.25) were prepared without pinning and magnetically coupled layers. Further, no postannealing was performed for the top Fe layer to keep it a general free layer and to limit the QW occurring only in the bottom ultrathin‐Fe layer. As well known, the dominant states of the electron transport in epitaxial MTJs with crystalline barrier consisting of MgO or MgAl_2_O_4_ possess the Δ_1_ (spd‐like) symmetry, while tunneling probabilities of other states, i.e., Δ_5_ (pd‐like), Δ_2_ (d‐like), and Δ_2′_ (d‐like) states, rapidly decay with increasing the barrier thickness.^4^, ^30^, ^31^ This is because electron coherence can be maintained in the tunneling process across the crystalline barrier with the Δ_1_ symmetry, resulting in the symmetry filtering. Thus, only Δ_1_ symmetry electrons need to be considered for these MTJs. In addition, the TMR effect is a resistance change depending on the relative angle of magnetization of the electrodes, and therefore the TMR ratio is given by the effective spin polarization of Δ_1_ electrons in the Fe electrodes. Since there is no Δ_1_ states near the Fermi level (*E*_F_) in Cr, even the metallic Cr layer can be regarded as a potential barrier in the present heterostructure of Cr/ultrathin‐Fe/MgAl_2_O_4_/Fe, leading to a quasi double‐barrier structure for the formation of QW states of Δ_1_ symmetry electrons (see Figure 1b,c). Consequently, the transport properties substantially depend on the applied *V*_bias_ via the energy levels of QWs. When *V*_bias_ is far from the resonant position, even the Δ_1_ symmetry electrons hardly contribute to the transport, as shown in Figure 1b. On the other hand, when *V*_bias_ coincides with the resonant position, the Δ_1_ electrons can pass through the corresponding QW state, as shown in Figure 1c. In fact, considering that the effective spin polarization of Δ_1_ electrons in Fe is near its *E*_F_, the quasi double‐barrier structure acts like a “spin filter” that efficiently enhances the spin polarization for the Δ_1_ symmetry electrons, as theoretically treated in ref. 10 for the Fe/MgO system. In this study, QW‐SDRT effect in MgAl_2_O_4_ is considered similar as in the MgO case, since the QW‐SDRT originates from imaginary band dispersions within the bandgap, where the MgAl_2_O_4_ shares nearly the same bandgap with MgO. More importantly, the disordered spinel structure obtained in this work can approximate to the MgO structure as discussed in a previous study.^32^

Owing to the interface effect of Fe/MgAl_2_O_4_,^33^, ^34^ the ultrathin Fe layer interface shows large perpendicular magnetic anisotropy (PMA), which leads to an orthogonal configuration of the easy magnetization axes of two Fe electrodes as shown in Figure 1. The bottom ultrathin‐Fe layer possesses an out‐of‐plane easy axis, while the shape magnetic anisotropy with an in‐plane easy axis is dominant in the top Fe electrode. Therefore, TMR was measured with an out‐of‐plane magnetic field, aligning the magnetization of top free Fe layer to the fixed bottom ultrathin Fe, as shown in Figure 1d. The observed TMR ratio of MTJs was defined as

(1) $$\mathrm{TMR}=\left(R_{90}-R_{P}\right)/R_{P}\times 100\left(\%\right)$$

where *R*_P_ and *R*_90_ are the tunnel resistance at saturation (i.e., parallel (P) magnetization configuration) and zero magnetic fields, respectively. Meanwhile, due to the orthogonal configuration at zero fields, the observed TMR is smaller than the conventional TMR that is calculated as

(2) $${\mathrm{TMR}}_{\mathrm{conventional}}=\left(R_{\mathrm{AP}}-R_{P}\right)/R_{P}\times 100\left(\%\right)$$

where *R*_AP_ is the tunnel resistance of antiparallel (AP) magnetization configurations. Since *R*_AP_ can be calculated from the relation^35^ of

(3) $$G_{90}=\left(G_{P}+G_{\mathrm{AP}}\right)/2$$

where *G*_P(AP)_ = 1/*R*_P(AP)_. The conventional TMR ratios are also calculated for comparison with the related results reported so far. We also investigated the differential conductance (d*I*/d*V*) spectra of the MTJs, since the resonant states show up as peak structures in the d*I*/d*V* spectra, as discussed in previous reports.^11^, ^17^, ^21^, ^22^, ^23^, ^24^

**Figure 2:**
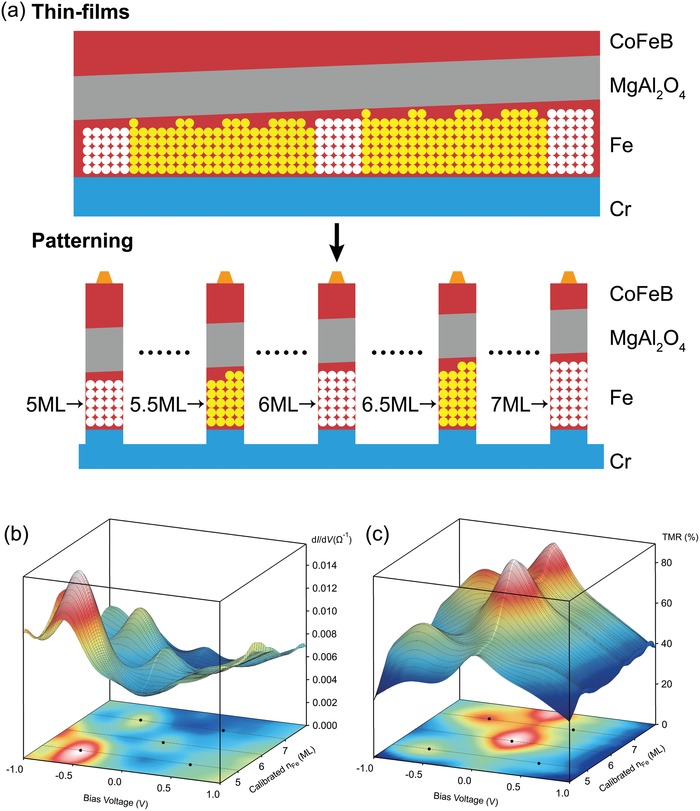
a) Thin films and patterned MTJs of Cr/ultrathin Fe/MgAl_2_O_4_/Fe with ultrathin wedge Fe layer. b) Conductance and c) TMR maps on bias voltage *V*_bias_ and calibrated Fe layer numbers *n*_Fe_ for patterned MTJs at RT. The resonant peak positions are marked with black points.

To prepare the MTJs with an exact integer number of Fe atomic layers, a wedge‐shaped Fe layer was deposited with the designed thickness from 0.45 to 1.45 nm using a linear motion shutter. This gradually increasing *t*_Fe_ ensured that MTJs with an integer number of Fe atomic layers existed in a certain part of the sample as **Figure** **2**a shows. Transport properties were measured for all the MTJs with different *t*_Fe_, and then the d*I*/d*V* spectra were summarized as a conductance map onto *V*_bias_ and *t*_Fe_ in Figure 2b, in which the peak structures clearly demonstrate the formation of QW states oscillatory with regard to the variation of *t*_Fe_. Here, it is considered that *t*_Fe_ at the peak positions correspond to integer numbers of Fe atomic layers. Due to shadow effect of a linear shutter, possible intermixing at the Cr/Fe interface, and/or the formation of FeO layer at the Fe/oxide interface,^24^ the effective Fe layer thickness, i.e., the QW width, may be smaller than *t*_Fe_. Based on theoretical calculations of QWs in the Cr/Fe/MgO case,^13^ the effective Fe layer thickness can be calibrated by the resonant peak positions (black points in Figure 2b), which is represented by the number of Fe atomic layers *n*_Fe_. Note that *t*_Fe_ ≠ *n*_Fe_ *d*_Fe_ due to discussion above, where *d*_Fe_ ≈ 0.143 nm is the thickness of a single Fe atomic layer, i.e., monolayer (ML). We attribute the peaks of *t*_Fe_ = 0.81, 1.02, and 1.22 nm to *n*_Fe_ = 5, 6, and 7 ML in this study by conductance spectrum estimation.

**Figure 3:**
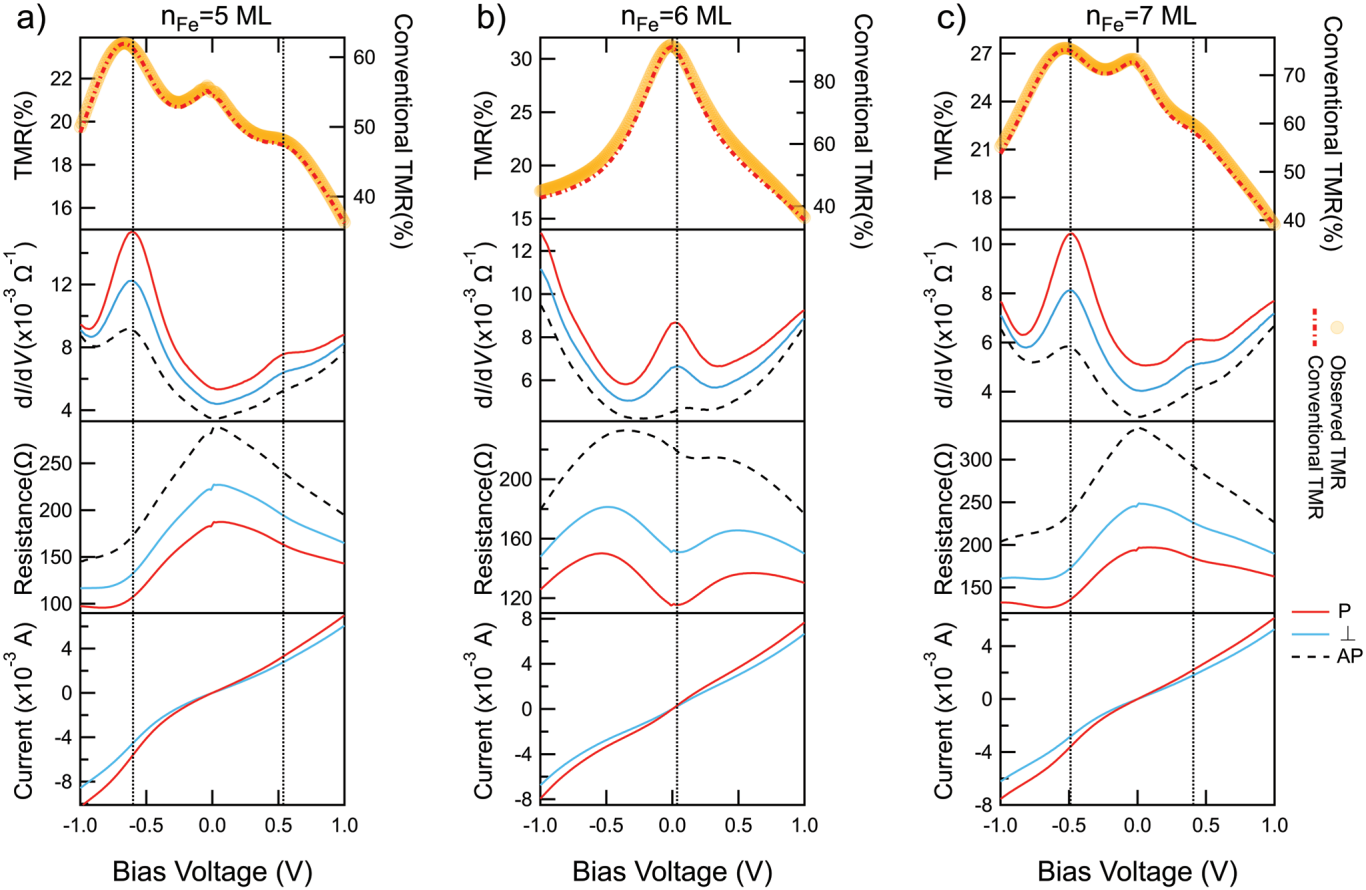
Bias voltage dependence of current, resistance, differential conductance, and observed‐TMR/conventional‐TMR (order from bottom to top) for MTJs with a) *n*_Fe_ = 5 ML, b) *n*_Fe_ = 6 ML, and c) *n*_Fe_ = 7 ML, respectively.

The TMR ratios are also summarized in the same manner as shown in Figure 2c. Comparing with the peak structures in the conductance map, the peaks in the TMR ratio map show the same period, which also indicates clearly that the TMR peaks originate from the SDRT effect. To discuss the SDRT in detail, the bias voltage dependences of TMR ratio (observed and conventional), d*I*/d*V*, resistance (*R*), and current for *n*_Fe_ = 5, 6, and 7 ML are compared in **Figure** **3**. In the d*I*/d*V* spectra, the even and odd *n*_Fe_ samples show clear difference, i.e., two resonant peaks arise away from zero bias for odd *n*_Fe_, while a single resonant peak arises around zero bias for the even *n*_Fe_. For *n*_Fe_ = 5 ML, the resonant peak positions are around *V*_bias_ = −0.58 and +0.55 V, as indicated with the dot lines in Figure 3a. The corresponding TMR peaks are observed at *V*_bias_, a little larger than those for d*I*/d*V*. The shift is presumably due to the fact that TMR at a given *V*_bias_ is calculated from the *R* values. The most important finding in Figure 3a is that the TMR peak of ≈24% at the resonant *V*_bias_ of −0.58 V is even higher than that of ≈22% at zero bias. This TMR‐enhanced behavior is in contrast to MgO‐based QW‐MTJs: TMR at resonant *V*_bias_ is 0.8 (≈0.6) times as large as that at zero bias at RT in Cr/Fe/MgO/Fe MTJ^21^ (Fe/MgO/Fe/MgO/Fe‐double‐barrier QW‐MTJ^25^). Only at very low temperature, a huge enhancement of TMR was reported so far.^20^ Moreover, since the resonant peak is existing at a high *V*_bias_ for odd *n*_Fe_, the TMR becomes less bias sensitive: TMR varies from ≈0.9 to ≈1.1 times of its zero bias value within [−1, 0 V], which is hardly found in conventional MTJs. Benefited from the enhanced TMR ratio at high bias voltage, i.e., a large output voltage is also obtained around 0.35 V at −1 V (see Figure S2 in the Supporting Information).

On the other hand, completely different behavior was observed in the MTJs with an even *n*_Fe_, showing a single TMR peak as normal MTJs. This is because the resonant *V*_bias_ is just located near zero bias, where the intrinsic TMR peak is normally located. For *n*_Fe_ = 5 ML, the TMR at *V*_bias_ = 0 is around 32%.

As mentioned above, the measured TMR ratios are smaller than the true values in their conventional definition due to the orthogonal configuration. Hence, d*I*/d*V* and *R* in the AP state are also deduced from those in the parallel (P) and 90° states to investigate the conventional TMR ratio. As shown in Figure 3, the SDRT effect on d*I*/d*V* in the AP states is much weakened, compared with in P states, which agrees well with the theoretical prediction mentioned above. To explore the possible performance in practical p‐MTJs, the conventional TMR calculated from *R* in the P and AP states is also plotted in Figure 3. For *n*_Fe_ = 5 ML, the TMR ratios at resonant and zero bias are 65% and 55%, respectively. For *n*_Fe_ = 6 ML, the TMR ratio at zero bias reaches 92%, which is comparable to the highest value in pure Fe‐based p‐MTJ.^33^ Since the top Fe electrode is not optimized at all in the present study, much higher TMR ratios are believed to be obtained for practical p‐MTJs made with optimization in the stack structure and preparation processes. In the case of noninteger *n*_Fe_, e.g., 5.5 and 6.5 ML, the peak structures in d*I*/d*V* and TMR behave as a simple sum of those with neighboring integer numbers of *n*_Fe_ (see Figure S1 in the Supporting Information). This suggests that it is possible to modulate the TMR behavior between single‐peak (for even *n*_Fe_ cases) type and multipeak (odd *n*_Fe_ cases) type simply by adjusting the Fe coverage.

The significant enhancement of TMR at resonant *V*_bias_, compared to the previous studies, is attributed to two major factors: improved structural coherency owing to MgAl_2_O_4_ and enhanced QW effect owing to the atomically flat ultrathin Fe layer. The former supplies a much‐improved high bias performance as previous works suggested.^27^ Inelastic electron scattering that causes suppression of TMR may occur hardly at the electrode/barrier interface free from misfit dislocations. For the latter one, both the transmission electron microscope image^27^ and the giant PMA observed in these films indicate the formation of atomically flat Fe/oxide interface,^36^ with which quantum interference can be increased. Besides, the thinner Fe thickness, e.g., effective QW width, is naturally leading to the enhancement of QW formation.

**Figure 4:**
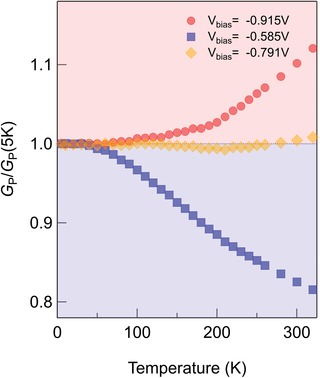
Temperature dependence of differential conductance of the parallel state at different bias voltages. *V*_bias_ = −0.915 V is out of the resonant range; *V*_bias_ = −0.585 V is just at the resonant bias; and *V*_bias_ = −0.791 V is in between.

**Figure 5:**
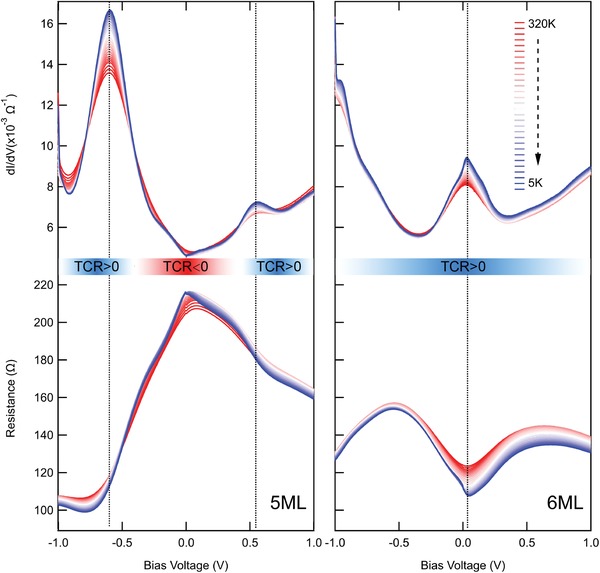
d*I*/d*V* and *R* (P‐state) as a function of *V*_bias_ for *n*_Fe_ of a) 5 ML and b) 6 ML. The color scale represents temperature changes from 5 to 320 K. The dotted lines show the resonant peaks and *V*_bias_.

It is well known experimentally that the tunneling transport of Δ_1_ symmetry electrons can presumably be characterized by temperature coefficient of resistance (TCR). In fact, tunneling transport normally shows negative TCR, whereas positive TCR in the P state has been observed for high‐quality Fe/MgO (MgAl_2_O_4_)‐based MTJs in which the Δ_1_ electron transport should be dominant.^5^, ^28^ The positive TCR is likely related to the dominance of the Δ_1_ preferential transport through Fe electrodes. **Figure** **4** shows the temperature dependence of differential conductance of parallel state (*G*_P_ = d*I*/d*V* at the P state) normalized by the 5 K values of the MTJ with *n*_Fe_ = 5 ML at selected bias voltages. A clear difference was observed among the temperature dependences of *G*_P_ at the bias voltages selected as around a resonant point (*V*_bias_ = −0.585 V), out of resonance (*V*_bias_ = −0.915 V) and in between the two (*V*_bias_ = −0.791 V). For the measurement around the resonant *V*_bias_, a clear negative correlation is observed, where *G*_P_ decreases as temperature increases, showing an elastic tunneling feature.^37^ On the other hand, for the measurement out of resonant *V*_bias_, a positive correlation, where *G*_P_ increases with temperature, is observed, showing an inelastic tunneling feature.^38^ Note that TCR in MTJs has been investigated based on elastic and inelastic tunneling processes. For a better comparison, d*I*/d*V* and *R* as a function of *V*_bias_ for *n*_Fe_ = 5 and 6 ML at different temperature are collected as **Figure** **5** shows. As the color scale from 5 K (blue) to 320 K (red) shows, unconventional positive TCR specific for the Δ_1_ electron transport is observed for *n*_Fe_ = 6 ML in the entire range of *V*_bias_. On the other hand, it is found that the sign of TCR changes when |*V*_bias_| exceeds the resonant positions shown by red arrows (*V*_bias_ < −0.58 V and *V*_bias_ > +0.52 V, as the blue arrows indicate). This phenomenon of TCR can be interpreted as follows: in the well‐defined QW‐MTJs, the Δ_1_ transport channels are highly localized around the resonant *V*_bias_, as suggested by the enhanced TMR appearing at the resonant *V*_bias_. As mentioned above, *R* at a given *V*_bias_ is given as the integration of the inverse d*I/*d*V* from 0 bias to the *V*_bias_, so that *R* at *V*_bias_ reflects d*I*/d*V* ranging from 0 bias to *V*_bias_. Therefore, for *n*_Fe_ = 5 ML, *R* hardly includes the Δ_1_ electron character below the resonant *V*_bias_, whereas above the resonant *V*_bias_, *R* turns to the resistance dominated by the Δ_1_ states, changing the sign of TCR. Positive TCR is always observed for *n*_Fe_ = 6 ML, since the resonant *V*_bias_ is around 0 V. As mentioned above, the observed behavior of TCR can be well interpreted as a fact that well‐defined QW states consisting of the Δ_1_ symmetry electrons are formed in the present Cr/Fe/MgAl_2_O_4_ structure, in short, as a signature of the well‐defined QWs.

## 3 Conclusions

In summary, we demonstrate the enhanced TMR by modifying the Δ_1_ electron tunneling transport via QWs that are definitely created in the Cr/ultrathin‐Fe/MgAl_2_O_4_/Fe MTJs. TMR at resonant *V*_bias_ is even higher than that at 0 bias at RT, while the behavior of d*I*/d*V* and TMR strongly depends on whether *n*_Fe_ is an even or odd number, suggesting that the SDRT through QWs is useful to achieve a large TMR, exclusively at high bias voltages. In addition, the sign change in TCR was discovered for the present MTJs, as evidence for the formation of well‐defined QWs.

## 4. Experimental Section

A fully epitaxial MgO (5 nm)/Cr (30 nm)/Fe‐QW (*t*_Fe_: 0.45−1.25 nm)/MgAl_2_O_4_ (2 nm)/Fe (10 nm)/Ru (15 nm) was prepared on a single crystalline MgO(001) substrate. All the layers, except sputtered Ru capping layer, were deposited by electron beam evaporation under a base pressure of less than 1 × 10^−8^ Pa. The substrate was first annealed at 800 °C for degassing and cleaning surface. Then the 5 nm MgO was deposited on substrate at 450 °C as a seeding layer. Then the Cr, QW‐Fe, and MgAl_2_O_4_ layers were deposited at 150 °C wherein the postannealing was performed at 800, 250, and 400 °C, respectively. The wedge‐shaped Fe layer was deposited with a step of 0.02 nm using a linear motion shutter equipped between the Fe source and substrate. The top Fe reference layer was deposited at RT without postannealing to prevent the formation of perpendicular anisotropy at the MgAl_2_O_4_/Fe interface. Finally, the stacks were capped with Ru layer via radio frequency magnetron sputtering with the process pressure of 0.4 Pa at RT without postannealing. The prepared film was patterned into ellipse junctions of 5 × 10 µm^2^ in size by a conventional microfabrication method using Ar‐ion etching and photolithography. Then, the junctions were measured for magnetoresistance and the current–voltage (*I–V*) curves using a dc four‐probe method, with a bias voltage up to ±1 V. The magnetic field up to 2 T was applied perpendicular to the film surface for RT measurement. A low‐temperature measurement was also performed using a physical property measurement system. The differential conductance (d*I*/d*V*) spectra were mathematically calculated from the *I–V* curves.

## Conflict of Interest

The authors declare no conflict of interest.

## Supporting information

Supplementary
