# Realizing Room‐Temperature Resonant Tunnel Magnetoresistance in Cr/Fe/MgAl_2_O_4_ Quasi‐Quantum Well Structures

**DOI:** 10.1002/advs.201902995

**Published:** 2019-11-20

**Authors:** Qingyi Xiang, Hiroaki Sukegawa, Mohamed Belmoubarik, Muftah Al‐Mahdawi, Thomas Scheike, Shinya Kasai, Yoshio Miura, Seiji Mitani

*Adv. Sci.* **2019**, *6*, 1901438

In the originally published article, the top layers of the thin films and patterned magnetic tunnel junctions (MTJs) in **Figure** **2** were incorrectly labeled as CoFeB. The correct Figure 2 is presented here. The authors apologize for any misunderstanding this may have caused.

**Figure 2:**
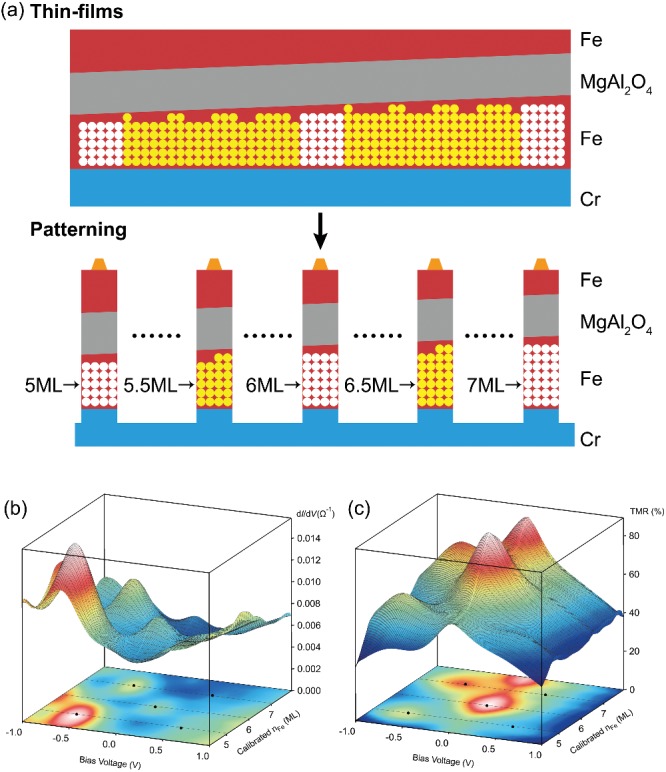
a) Thin films and patterned MTJs of Cr/ultrathin Fe/MgAl_2_O_4_/Fe with ultrathin wedge Fe layer. b) Conductance and c) TMR maps on bias voltage *V*_bias_ and calibrated Fe layer numbers *n*_Fe_ for patterned MTJs at RT. The resonant peak positions are marked with black points.

