# Supplementary material for: Realizing Room‐Temperature Resonant Tunnel Magnetoresistance in Cr/Fe/MgAl2O4 Quasi‐Quantum Well Structures
Source: Adv Sci (Weinh). 2019 Aug 10;6(20):1901438. doi: 10.1002/advs.201901438 (PMC6794625; doi:10.1002/advs.201901438)
Supplement: Supplementary file 1 — Supplementary [file ADVS-6-1901438-s001.pdf]

Copyright WILEY-VCH Verlag GmbH & Co. KGaA, 69469 Weinheim, Germany, 2019.

ADVANCED SCIENCE Open Access

Supporting Information

for *Adv. Sci.,* DOI: 10.1002/advs.201901438

Realizing Room-Temperature Resonant Tunnel Magnetoresistance in Cr/Fe/$MgAl_2O_4$ Quasi-Quantum Well Structures

Qingyi Xiang, Hiroaki Sukegawa, Mohamed Belmoubarik, Muftah Al-Mahdawi, Thomas Scheike, Shinya Kasai, Yoshio Miura, and Seiji Mitani**

Supporting Information

Realizing Room Temperature Resonant Tunnel Magnetoresistance in Cr/Fe/$MgAl_2O_4$ Quasi Quantum Well Structures

Q.Y. Xiang, H. Sukegawa, M. Belmoubarik, M. Al-Mahdawi, T. Scheike, S. Kasai, Y. Miura, and S. Mitani**

1. Quantum well state transition: from one integer monolayer to another integer monolayer

In this study, to preciously get an integer monolayer number of Fe for obtaining high quantity quantum wells, a wedge Fe layer with a limited thickness variation is prepared by EB-evaporation. The sample is fabricated into many MTJs with the t_{Fe} step of 0.02 nm, and we successfully obtained MTJs with exactly integer monolayer numbers and those with non-integer monolayer numbers. Figure S1 shows selected dI/dV spectra and TMR curves from 5 ML to 7 ML, including two non-integer monolayer number samples, 5.5 ML and 6.5 ML. It is clearly seen how the quantum well states cycle with the t_{Fe} varies. As Figure S1 shows, the features of 5.5 ML sample are actually the mixture of 5 ML and 6 ML samples, which agrees well with the definition of the non-integer layer.

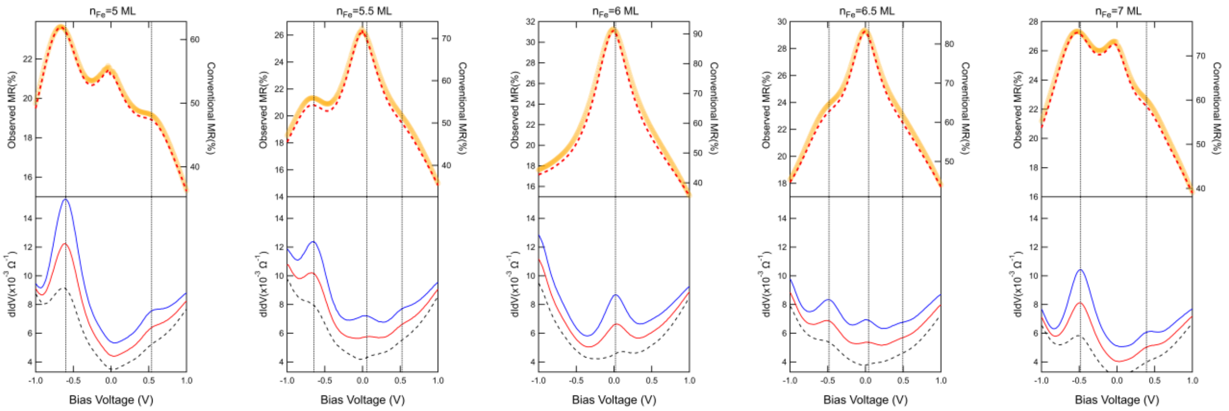

2. Enhanced output voltage

Output voltage is important for both sensor and MRAM applications. In this study we calculated the V_{output} with: $V_{output} \equiv (R_{AP} - R_{P}/R_{AP}) \times V_{bias}$. As Figure S2 shows, the V_{output} is significantly enhanced at the high bias range, reaching 0.35 V at –1 V, which is the highest value for the MTJ with perpendicular free layer. The enhancement of V_{output} is attributed to the existing QW states in the high bias voltage range, in which TMR enhances. Meanwhile, the enhanced TMR at the high bias voltage makes the TMR ratio stable in a wide range of the bias, which leads to a great improvement of the linearity of the output voltage as Figure S2 shows. Note that the solid red line is reproduced V_{output} from Ref. 29, which was the reported highest TMR ratio in p-MTJs previously. The largest V_{output} reproduced is around 0.27 V around –0.6V.

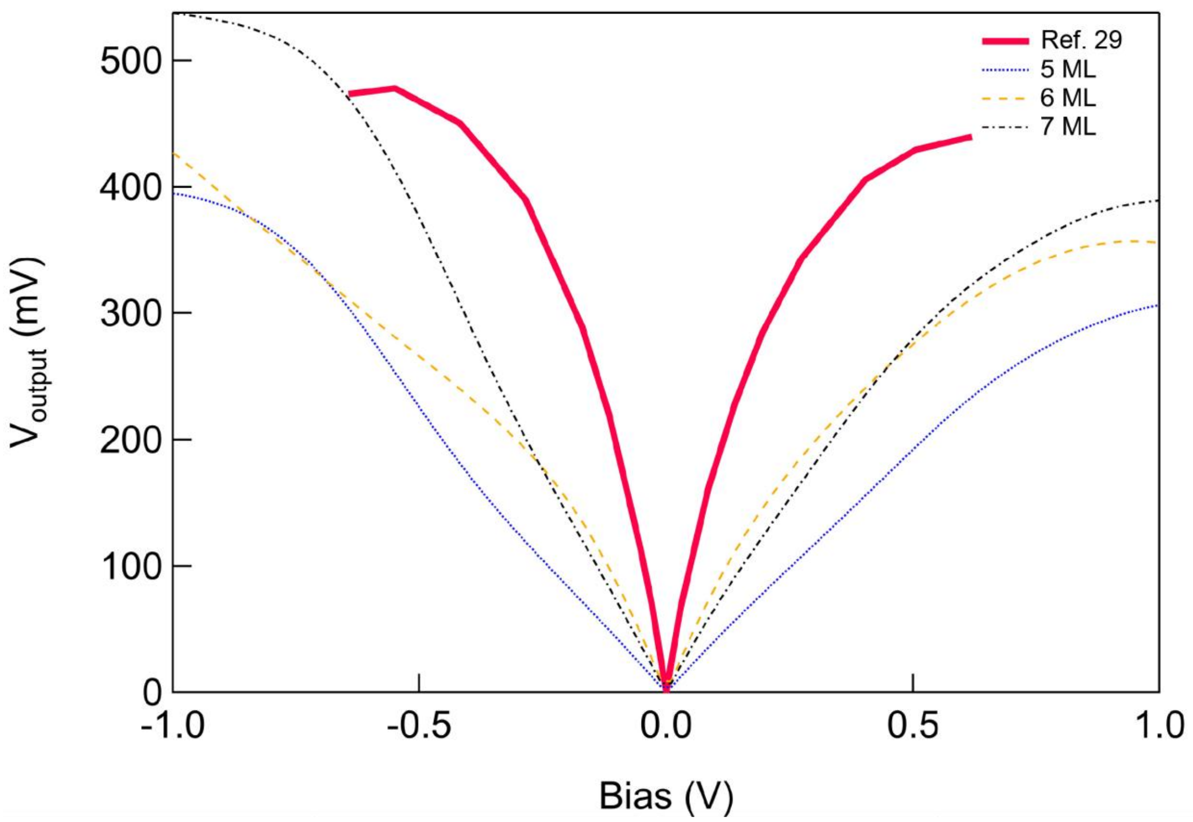
Ref. 29
5 ML
6 ML
7 ML
V_{output} (mV)
Bias (V)
500
400
300
200
100
0
-1.0
-0.5
0.0
0.5
1.0
